# Mastering your fellowship: Part 2, 2023

**DOI:** 10.4102/safp.v65i1.5720

**Published:** 2023-04-24

**Authors:** Mergan Naidoo, Klaus von Pressentin, Tasleem Ras, Joyce Musonda

**Affiliations:** 1 Department of Family Medicine, College of Health Sciences, University of KwaZulu-Natal, Durban, South Africa; 2 Department of Family, Community and Emergency Care, Faculty of Health Sciences, University of Cape Town, Cape Town, South Africa; 3 Department of Family Medicine, Faculty of Health Sciences, University of Pretoria, Pretoria, South Africa

**Keywords:** family physicians, FCFP (SA) examination, family medicine registrars, postgraduate training, national exit examination, child health

## Abstract

The ‘Mastering your Fellowship’ series provides examples of the question format encountered in the written and clinical examinations, Part A of the Fellowship of the College of Family Physicians of South Africa (FCFP [SA]) examination. The series is aimed at helping family medicine registrars prepare for this examination.

## Introduction

This section in the *South African Family Practice* journal is aimed at helping registrars prepare for Part A of the Fellowship of the College of Family Physicians of South Africa (FCFP [SA]) examination and will provide examples of the question formats encountered in the written examination: multiple choice question (MCQ) in the form of single best answer (SBA – Type A) and/or extended matching question (EMQ – Type R); short answer questions (SAQs), questions based on the critical reading of a journal (evidence-based medicine) and an example of an objectively structured clinical examination (OSCE) question. Each of these question types is presented based on the College of Family Physicians blueprint and the key learning outcomes of the FCFP programme. The MCQs are based on the 10 clinical domains of family medicine, and the SAQs will be aligned with the five national unit standards. The critical reading section will include evidence-based medicine and primary care research methods.

This edition is based on unit standard one (critically reviewing new evidence and applying the evidence in practice, leadership and governance), unit standard two (evaluate and manage a patient according to the biopsychosocial approach) and unit standard three. (Improve the health and quality of life of the community.) The domain covered in this edition is child health. We suggest you attempt to answer the questions (by yourself or with peers or supervisors) before finding the model answers online: http://www.safpj.co.za/.

Please visit the Colleges of Medicine website for guidelines on the Fellowship examination: https://www.cmsa.co.za/view_exam.aspx?QualificationID=9.

We are keen to hear about how this series assists registrars and their supervisors in preparing for the FCFP (SA) examination. Please email us (naidoom@ukzn.ac.za) your feedback and suggestions.

## Multiple choice question (MCQ): Single best answer

During March 2022 and April 2022, many children under 2 years of age were seen in the emergency centre (EC) with fever, cough and dyspnoea. Accompanying signs included pyrexia, tachypnoea, tachycardia, bilateral wheeze, with or without crepitations and hypoxia. Chest X-ray revealed an infiltrative pattern. They often needed admission to the hospital. The staff are worried about this potential outbreak. What is the most appropriate next investigation?

- Blood cultures
- Microscopy and culture of sputum
- SARS-CoV-2 antigen test
- SARS-CoV-2 polymerase chain reaction (PCR) test
- Viral multiplex swab

*Answer: e*

Amid the coronavirus disease 2019 (COVID-19) pandemic in early 2022, we experienced an influx of young children presenting with respiratory distress, as outlined here. These children were often very ill and needed admission. Most were fully immunised and had no other comorbidities. The hospital’s routine practice was to perform severe acute respiratory syndrome coronavirus-2 (SARS-CoV-2) antigen tests on these children, but these tests were all negative. Coronavirus disease 2019 was not known to cause severe disease in healthy young children. A strong suspicion was that this was a rebound of respiratory viruses that had been somewhat suppressed during the first 2 years of the pandemic because of infection prevention and control (IPC) practices implemented nationally. The National Institute of Communicable Diseases issued an alert to clinicians in late March 2022 warning of a potential outbreak of the respiratory syncytial virus (RSV), which was suppressed because of non-pharmacological interventions in 2020 and 2021. The anticipated autumn/winter peaks of RSV in children under five did not materialise. The alert further warned that RSV was detected in 19% of respiratory pathogens in March 2022. The potential RSV resurgence in 2022, age-structured epidemiological models were aligned to the national surveillance data from South Africa and predicted the 2022 RSV outbreak following the 2 years of low RSV transmission. The models predicted an overall 32% increase in the peak number of monthly hospitalisations compared with the average for 2015–2019.

It is always helpful to consult with relevant specialists, particularly paediatric infectious disease specialists when investigating respiratory disease outbreaks in children. The surveillance data from the National Institute of Communicable Diseases (NICD) are also beneficial, but the local investigation is essential to confirm that the local patterns fit into the epidemiologic predications. Appropriate involvement of multidisciplinary teams is necessary for planning. These include IPC managers, institutional and district levels, and staff managing patients. The institution’s management also needs to be appraised of the situation, and the referral centre needs to be informed. A reporting and monitoring system needs to be implemented, and the services may need to be modified to deal with the outbreak. In this case, the Kangaroo Mother Care was temporarily suspended at the institution as it was deemed to be too risky for premature neonates. The outbreak lasted about 2 months, and the last child with RSV bronchiolitis was discharged in mid-May 2022.

Respiratory syncytial virus causes bronchiolitis and is associated with an increased incidence of asthma in children presenting with recurrent episodes. Most cases are mild and are treated symptomatically, but sometimes children present with severe respiratory distress and hypoxia warranting oxygen therapy and admission. Severe cases may require admission to the intensive care unit. Once the diagnosis is confirmed oxygen and hypertonic saline nebulisation are used for treatment. Most children gain passive immunisation from their mothers for the first 4 months of life and become vulnerable after that. There is currently no available vaccine. Chest X-rays are usually not helpful in diagnosing. Still, the Rapid Multiplex PCR Assay is a respiratory panel that can efficiently test for various viruses, including SARS-CoV-2, RSV, influenza and parainfluenza. It is also relatively cheap and available through the National Health Laboratory Service.

**Further reading**

- National Institute of Communicable Diseases. RSV alert for healthcare workers [homepage on the Internet]. NICD Bulletin. 23 March 2022 [cited 2022 November 20]. Available from: https://www.nicd.ac.za/wp-content/uploads/2022/03/RSV-season-has-started_March-2022_Final.pdf
- Mammas IN, Drysdale SB, Rath B, et al. Update on current views and advances on RSV infection. Int J Mol Med. 2020;46(2):509–20. https://doi.org/10.3892/ijmm.2020.4641
- Newman H, Tshabalala D, Mabunda S, Nkosi N, Carelson C. Rapid testing for the respiratory syncytial virus in a resource-limited paediatric intensive care setting. Afr J Lab Med. 2020;9(1):1–5. https://doi.org/10.4102/ajlm.v9i1.1084

## Short answer question (SAQ): The family physician’s role as a champion of community oriented primary care

You are the family physician working at a community health centre. The Ward-Based Outreach Team leader informs you of an increase in severe acute malnutrition in children under five in the community. You decide to assist with this issue in the community:

1. What important information and data will you utilise to describe and explain this health problem in the community? (5 marks)
2. What further steps in the community-orientated primary care approach will you now to take to assist with this issue? (5 marks)
3. How will you ensure that the care of children admitted with severe acute malnutrition (SAM) in the hospital is coordinated on discharge? (5 marks)
4. List five broader interventions you will use to address malnutrition in children in this community. (5 marks)

Total: 20 marks

Model answers

### 1. What important information and data will you utilise to describe and explain this health problem in the community? (5 marks)

*(Any 5 of the following points in each of the different areas)*

Local health analysis:

- Demographics: Measure the population of under-five children in the community from ward based outreach team (WBOT) household registrations *or* information about migration – WBOT to conduct home visits to identify vulnerable/undocumented children who may not be entitled to social services, for example, food security and child grant.
- Health status: Morbidity profile – Establish how many under five children have severe acute malnutrition and the commonest causes *or* Measure how many under-five children are being diagnosed with SAM at the community healthcare centre (CHC) level *or* utilise WBOT team leaders and community health workers (CHWs) to identify children in the community that have malnutrition but have not yet sought medical help. Mortality profile – Get information on case fatality rates from malnutrition within different age groups from District Health Information Systems.
- Socio-economic: Poverty level – Get information on the poverty level in the community from WBOT household assessment and/or statistics South Africa to measure the poverty level of the community *or* obtain information about the unemployment rate, literacy rate and level of education, housing.
- Health services: Utilise WBOT to identify vulnerable children (undocumented or those whose parents are alcoholics) *or* utilise WBOT to identify mothers that are not accessing health services and what their reasons are *or* audit information at the CHC level from mothers to whether health services are accessible.
- Alternative health services: Build relationships with the community in order to find out about their beliefs, attitudes and utilisation of the health services and disease conditions – malnutrition *or* collaborate and train local traditional healers to identify and refer such children immediately to the CHC.
- Lifestyle and habits: Measure the problem of alcoholism and substance abuse in the community, as well as identifying potentially unsupported families such as single mothers or child-headed households in the community.

### 2. What further steps in the community-orientated primary care approach will you now to take to assist with this issue? (5 marks)

- Join the healthcare team responsible for that community, for example, a ward health team and/or form a team of relevant stakeholders.
- Do a local institutional analysis of available resources in that community. Community health forum can bring all the role players together.
- Prioritise health needs: Health needs are analysed to identify the most important one which is prioritised.
- Create adaptive action plans and implement interventions to address the individual, household, community health issues.
- Monitor activities and plans, evaluate, review, reflect, reprioritise, replan, react.

### 3. How are you going to ensure that the care of children admitted with SAM in the hospital is coordinated on discharge? (5 marks)

*(1 mark each for any 5 of the following points)*

- Link members of the WBOT teams to the hospital to facilitate coordination of care.
- Conduct multidisciplinary ward rounds to facilitate coordination of care between facility- and community-based staff members.
- All discharged SAM cases to be linked to the local primary health care team nurse, doctor and WBOT.
- All discharged SAM cases to be linked to a local dietician so that they are closely monitored.
- Ensure ongoing household visits of discharged SAM cases to report on progress *or* defaulting / not gaining weight *or* losing weight on follow-up visit *or* child has returned to inpatient care *or* refuses a referral to inpatient care.
- All discharged SAM cases to be linked to local social services for food security. All discharged SAM cases to be linked to local social workers who will assess the home environment.

### 4. List five broader interventions you will use to address malnutrition in children in this community. (5 marks)

*(1 mark each for a ny 5 points from the given list)*

- Train WBOT team leaders/CHWs on how to identify children who are at risk of malnutrition.
- WBOT team leaders/CHWs to conduct campaigns and awareness for malnutrition in the community.
- Health promoters and team leaders to offer child nutrition information to the community.
- Offer child nutrition information to mothers and care givers.
- Train WBOT and CHWs on how to assess and measure mid-upper arm circumferences (MUACs) for all children.
- Assess children’s growth charts in their road to health books (RTHBs).
- Identify children not on child grant and refer them to social services.
- Identify children with food insecurity and link them to local social services and non-governmental organisations for food parcels.

**Further reading**

- Mash B, Blitz-Lindeque J. South African family practice manual. 2nd ed. Pretoria: Van Schaik Publishers 2006.
- Mash R, Gaede B, Hugo JF. The contribution of family physicians and primary care doctors to community-orientated primary care. S Afr Fam Pract. 2021;63(1):e1–e5. https://doi.org/10.4102/safp.v63i1.5281

## Critical appraisal of research

Read the accompanying article carefully and then answer the following questions. As far as possible use your own words. Do not copy out chunks from the article. Be guided by the allocation of marks concerning the length of your responses.

- Besselink D, Brandt H, Klingberg S, Draper CE. Perceptions of healthy food, and perceived facilitators and barriers to buying and consuming healthy food, among female caregivers in Soweto, South Africa. S Afr J Child Health. 2022;16(3):172–177. https://doi.org/10.7196/SAJCH.2022.v16i3.1883.

1. Did the study address a clearly focused question? Discuss. (2 marks)
2. Explain why a qualitative research methodology may be most appropriate for this research question. Comment on the authors’ choice of qualitative study design. (5 marks)
3. Critically appraise the participant recruitment and sampling strategy. (5 marks)
4. Critically appraise the choice of instruments as described in the methods section. (4 marks)
5. Critically appraise the reporting of reflexivity (researcher positionality) in the paper. (5 marks)
6. Were the participants included in the study well described? Justify your response. (3 marks)
7. Use a structured approach (e.g. relevance, education, applicability, discrimination, evaluation, reaction [READER]) to discuss the value of these findings to your practice. (6 marks)

Total: 30 marks

Model answers

**FIGURE 1:**
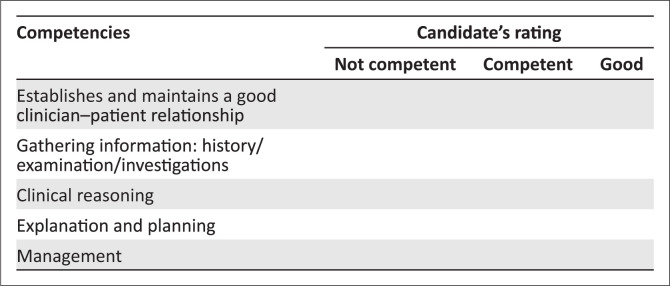
Marking template for consultation station.

1. **Did the study address a clearly focused question? Discuss. (2 marks)**
  - The authors aimed to provide an in-depth understanding of female caregivers’ perceptions of healthy food in an urban township with a focus on female caregivers of 3–5-year-old children.
  - The question is focused as it describes the population of interest (female caregivers of 3–5-year-old children), and the condition or phenomenon of interest (female caregivers’ perceptions of healthy food) in a particular community or area (an urban township).
2. **Explain why a qualitative research methodology may be most appropriate for this research question. Comment on the authors’ choice of qualitative study design. (5 marks)**
  - Qualitative research aims to understand meaning, such as the meanings that people attribute to their work, their behaviours or beliefs, or their attitudes or perceptions.
  - For this study, the authors hoped to obtain an in-depth understanding of a specific context in which future interventions, which target obesity-related behaviours may be implemented. They wished to understand the attitudes or perceptions of these female caregivers regarding healthy food.
  - This supports the choice of using a qualitative approach compared with a quantitative approach, which aims to develop objective theories by generating quantifiable numerical data.
  - The authors described their qualitative study design as exploratory using individual semi-structured interviews, to allow participants to elaborate on their experiences and feelings about healthy food. Exploratory studies aim to generate new knowledge by exploring novel topics where little or no data exists. These exploratory study designs include quantitative (surveys) as well as qualitative research methods (participant observation, interviews and focus groups).
  - In this article, the authors did not state their philosophical perspective and its alignment with their choice of research methodology. Most qualitative studies are descriptive in nature, and it would have been good to report the chosen methodology, for example, phenomenology, grounded theory, ethnography and narrative inquiry. The consolidated criteria for reporting qualitative studies (COREQ) state that the methodological orientation should be stated to underpin the study.
3. **Critically appraise the participant recruitment and sampling strategy. (5 marks)**
  - The researchers employed a type of purposive sampling method called criterion sampling, as they used four inclusion criteria to choose the participants who will potentially be the most informative in terms of their ability to answer the study question.
  - Three of the four inclusion criteria make sense in terms of the study’s aim, namely being a female caregiver of a child between approximately 3 and 5 years old, living in Soweto, and having a minimum age of 18 years. The minimum age of the respondents reduces the ethical complexity of the research project (especially obtaining consent), and one may argue that the views of teenage female caregivers would have warranted a separate study.
  - However, the willingness and ability to be interviewed in English may have excluded potential participants who may be more representative in terms of the researchers’ interest in understanding the specific context of black African households. This is ironic as the authors stated that they received translation assistance from a local research assistant during the interviews where necessary, as this means that they were prepared to interview non-English speaking participants.
  - The authors identified potential participants from an existing dataset compiled during a household survey, which contained a list of individuals who had consented to be re-contacted for future studies. This may represent a risk of respondent burden or fatigue if the same participants are asked to be included in subsequent studies. Fortunately, this existing list was used for screening purposes only and did not replace informed consent.
  - The authors stated that 10 participants were recruited but did not justify this decision. Usually, for individual interviews, a sample size of between 5 and 15 interviewees is adequate to obtain a sufficient range of responses and experiences, until a level of so-called data ‘saturation’ is reached. It is important to include the rationale for determining how one will gauge whether sufficient participants have been interviewed to meet the study objectives. The authors also did not provide information on the number of screened individuals who declined to participate, as well as their reasons for refusal.
4. **Critically appraise the choice of instruments as described in the methods section. (4 marks)**
  - The authors stated that the interview guide for the individual semi-structured interviews was developed to allow participants to elaborate on their experiences and feelings about healthy food. Two conceptual frameworks were used to guide the scope of the questions included in the guide. The first framework, ‘Attitude, Social Influence and Self-Efficacy model’, was used to capture personal factors influencing food choices. The second framework, the socio-ecological model (SEM), was used to capture external factors influencing food choices. The frameworks were not included in the article.
  - It is not clear from the article how these two frameworks were used to develop the interview guide in terms of adaption, translation and validation for the local South African setting. Based on the list of references, the authors cited three articles, which studied Ecuadorian adolescents, low-income African Americans and low- and middle-income country patients accessing clubfoot treatment, respectively. These patient populations appear quite dissimilar from the participants of this study.
  - The authors stated that ‘after a pilot interview and throughout the interview process, questions were added and removed and adaptions were made to make the questions easier to understand’. It is not clear how the interview guide was adapted and administrated across the 10 interviews by the research assistant – the authors did state that ‘the strengths of the study lay in the continuous discussion between the researcher and the research assistant’ and link this ongoing interaction with an assurance of the credibility of the study’s findings. However, the authors did mention the study’s limitations related to the risk of ‘potential misinterpretations owing to translation and cultural differences’.
  - Admittedly, one may argue that the process of adaption, translation and validation of an interview guide need not follow the same rigour as a quantitative survey instrument. Nonetheless, this section in the methods does not help the reader with gauging the process of developing this guide from the two frameworks mentioned, demonstrating the relevance of these frameworks in the local study setting and confirming whether all 10 participants were asked the same set of guiding questions. At the very least, the interview guide should have been included in the article or as a supplemental file.
5. **Critically appraise the reporting of reflexivity (researcher positionality) in the article. (5 marks)**
  - The article does not contain a reflexivity report or statement. The personal characteristics of the researcher(s) and research assistant(s) need to be reported to help the reader understand their positionality in terms of the research question, setting and participants. In the ‘Author contributions’ section, it is stated that ‘DB collected and analysed the data, with input from co-authors’. These initials refer to the first author of the article, and we know that a ‘local research assistant’ was involved in the interviews, which took place in the participants’ homes. The gender of the research team members is also unknown.
  - The credentials of the first author and research assistant are unknown. Do they have a medical or health sciences background? The researchers’ occupation at the time of the study is not provided. The highest degree of the research team members is provided (Masters in Medical Sciences [MMedSci] and Doctor of Philosophy [PhD]), which represent a combination of research degrees.
  - Two of the authors are affiliated with a Dutch University and two of the authors are affiliated with a health research unit at a South African University. The South African University shares the same setting as the research participants, but it is not described how the two Dutch authors are related to the research project. The same South African university conducted the household survey, which provided the list of individuals from which the study participants were recruited. It is stated that the first author from the Dutch university made the initial telephonic contact with the potential participants.
  - The experience and training of the research team, including the researcher assistant, are not described in sufficient detail. It is important to state the experience and training of the team members who designed the study, conducted the interviews, and conducted the data analysis. The research assistant is merely described as being ‘local’ and this assistant helped with translation where necessary.
  - The research team consists of a combination of Dutch and South African team members with undefined research backgrounds. More information on reflexivity should have been provided to locate the researchers culturally and theoretically. This information on the personal characteristics of the research team helps the reader to gauge the credibility of the researchers, as well as their reasons for doing the research. It will also help to clarify the existing relationships between the researchers and participants, to help the reader understand the researchers’ orientation to the local lived reality and cultural worldviews of the participants, in order to illuminate any potential risk for bias and assumptions.
6. **Were the participants included in the study well described? Justify your response. (3 marks)**
  - The 10 participants’ demographic information is presented in Figure 1 (Table 1 in Besselink et al 2022) of the article. Information about their age, as well as the age of their child, is available for most of the participants, which helps the reader to learn that the participants are between the ages of 37 and 44 years and are caring for children between the ages of 2 and 7 years. Eight participants are the primary caregivers, half of the participants are unmarried and only two participants indicated that they were married. Most participants receive a social grant. No information on home language(s) is provided.
  - In summary, the included participants are well described by the information presented in the table. The information helps the reader to contextualise the findings: the participants are in their late 30s or early 40s and largely unmarried, unemployed and receiving social grants. However, the ages of the two non-primary caregivers are not provided and no information is provided on the potential participants who were screened telephonically. Furthermore, the study targeted female caregivers of 3–5-year-old children, whereas the age range of the children of the participants extended to the ages of 2 and 7 years.
  - The demographic information of the Sowetan community is not provided in the study setting description, which makes it difficult to assess how representative these 10 participants are. No link or reference is provided to the previous household survey, which may also have assisted the reader with gauging the generalisability of the study findings.
7. **Use a structured approach (e.g. relevance, education, applicability, discrimination, evaluation, reaction [READER]) to discuss the value of these findings to your practice. (6 marks)**
  - The READER format may be used to answer this question:
    ■ Relevance to family medicine and primary care?
    ■ Education – does it challenge existing knowledge or thinking?
    ■ Applicability – are the results applicable to my practice?
    ■ Discrimination – is the study scientifically valid enough?
    ■ Evaluation – given the aforementioned, how would I score or evaluate the usefulness of this study for my practice?
    ■ Reaction – what will I do with the study findings?
  - The answer may be a subjective response but should be one that demonstrates a reflection on the possible changes within the student’s practice within the South African public healthcare system. It is acceptable for the student to suggest how their practice might change, within other scenarios after graduation (e.g. private general practice). The reflection on whether all important outcomes were considered is, therefore, dependent on the reader’s perspective (is there other information you would have liked to see?).
  - A model answer could be written from the perspective of the family physician employed in the South African district health system:
    ■ R: This study is relevant to the African primary care context, as obesity is a well-established public health concern. A better understanding of the underlying sociocultural influences on nutrition choices in low socio-economic settings will help primary care teams and policymakers to plan appropriate interventions.
    ■ E: The authors explored the perceptions of female caregivers of healthy food in an urban township, with a focus on female caregivers of 3–5-year-old children. Little is known about the motives behind food choices when considering the healthfulness of food in low-income households in South Africa, particularly in relation to the healthy eating habits of young children.
    ■ A: For this study, it would be possible to generalise the study’s findings to a similar South African urban township setting.
    ■ D: In terms of discrimination, there is a fair congruity between the research methodology and the data collection methods, as well as the analysis of data (it would have been helpful to state the methodological position more clearly as well as provide more information on how the two frameworks were used to develop the interview guide). The lack of stated reflexivity on the part of the research team impacts the reader’s assessment of confirmability (objectivity). Furthermore, the researchers did not perform participant checking or verification of returned transcripts which may impact the trustworthiness of the study findings.
    ■ E: The study’s findings may be relevant to consider when planning possible home-based health interventions in a similar setting as part of interdisciplinary community-orientated primary care. Some of the study findings did point to the role of community health workers operating from local health clinics, as well as the need to assess the accessibility of healthy eating guidance provided. Financial considerations are essential to ensure the affordability of healthy food. It may be useful to remind oneself of the study limitations, as well as that the study setting was that of an urban environment.
    ■ R: The study’s findings may be useful to reflect on when reviewing possible community-level interventions by the primary care team in partnership with the community-based stakeholders. As the study was exploratory in nature with several methodological concerns, however, its potential to influence behaviour in clinical practice is restricted.

**Further reading**

- Williams V, Boylan AM, Nunan D. Critical appraisal of qualitative research: necessity, partialities and the issue of bias. BMJ Evidence-Based Med. 2020;25(1):9–11. https://doi.org/10.1136/bmjebm-2018-111132
- Tong A, Sainsbury P, Craig J. Consolidated criteria for reporting qualitative research (COREQ): A 32-item checklist for interviews and focus groups. Int J Qual Health Care. 2007;19(6):349–357. https://doi.org/10.1093/intqhc/mzm042
- Reid S, Mash B. African primary care research: Qualitative interviewing in primary care. Afr J Prim Health Care Fam Med. 2014;6(1):1–6. https://doi.org/10.4102/phcfm.v6i1.632
- Mabuza LH, Govender I, Ogunbanjo GA, Mash B. African primary care research: Qualitative data analysis and writing results. Afr J Prim Health Care Fam Med. 2014;6(1):1–5. https://doi.org/10.4102/phcfm.v6i1.640
- The Joanna Briggs Institute. JBI QARI Critical appraisal checklist for interpretive & critical research. Adelaide: The Joanna Briggs Institute; 2014 [cited 2022 Nov 28]. Available from: https://jbi.global/sites/default/files/2019-05/JBI_Critical_Appraisal-Checklist_for_Qualitative_Research2017_0.pdf
- MacAuley D. READER: An acronym to aid critical reading by general practitioners. Br J Gen Pract. 1994;44(379):83–85.

## Objectively structured clinical examination (OSCE) scenario

Objectively structured clinical examination station: Child Health

### Objective of the station

This station tests the candidate’s ability to manage a paediatric patient with chronic constipation.

### Requirements

- Simulated patient: adult male/female

### Instructions for candidate

- You are the family physician working at a district hospital. On the ward round, you are asked to see a 3-year-old girl with faecal incontinence.

### Your task

- Please consult with this patient and develop a comprehensive management plan
- You do not need to do an examination on this patient. All examination findings will be provided on request

### Instructions for the examiner

This is an integrated consultation station in which the candidate has 15 min. Familiarise yourself with the assessor guidelines, which details the required responses expected from the candidate.

No marks are allocated. In the mark sheet (Figure 1), tick off one of the three responses for each of the competencies listed. Make sure you are clear on what the criteria are for judging a candidates’ competence in each area.

**Further reading**

- Department of Health, South Africa. 2.2.2 Constipation/Faecal loading. Standard treatment guidelines and essential medicines list hospital level: Paediatrics. Pretoria: South African National Department of Health, 2017.
- Division of Clinical Pharmacology and South African Medical Association. South African medicines formulary. 13th ed. Pretoria: Health and Medical Publishing Group; 2020, p. 59–62.

### Guidance for examiners

The aim is to establish that the candidate can diagnose chronic constipation in a child, identify possible causes (dietary, lifestyle, consider underlying medical conditions), and develop an effective and safe management plan.

*Not competent*: Patients’ safety is compromised (including ethico-legal aspect) or task is not completed.

*Competent*: The task is completed safely and effectively.

*Good*: In addition to displaying competence, the task is completed efficiently and in an empathic, patient-centred manner (acknowledges patients’ ideas, beliefs, expectations, concerns/fears).

#### Establishes and maintains a good clinician–patient relationship

The competent candidate is respectful, engaging with the patient in a dignified manner.

The good candidate is empathic, compassionate and collaborative, facilitating patient participation in key areas of the consultation.

#### Gathering information

The competent candidate gathers sufficient information to establish a diagnosis (*faecal incontinence secondary to chronic constipation with faecal loading, explores self-medication or home remedies*) and the parent’s concerns of *an underlying surgical problem*.

The good candidate additionally has a structured and holistic approach (*enquiring about other children, patterns of bowel movement since birth, breastfeeding, and current dietary patterns*).

#### Clinical reasoning

The competent candidate identifies the diagnosis (*faecal incontinence secondary to chronic constipation, most likely because of low dietary fibre intake*), and may acknowledge *some of the parent’s concerns*.

The good candidate makes a specific diagnosis (*chronic constipation because of poor fibre intake*) and has a structured approach to addressing psychosocial aspects (*parental anxiety of an underlying surgical problem and possible damage as a result of injudicious use of laxative; mentions other medical problems that must be ruled out*).

#### Explaining and planning

The competent candidate uses clear language to explain the problem to the patient and uses strategies to ensure patient understanding (*questions or feedback or reverse summarising*).

The good candidate additionally ensures that the patient is actively involved in decision-making, paying particular attention to knowledge-sharing and empowerment and *re-assures the parent’s concerns about iatrogenic harm done to the child.*

#### Management

The competent candidate proposes appropriate intervention (*dis-impaction with enema* [*sodium phosphate or glycerol*] *preceded by topical anaesthetic or polyethylene glycol orally immediately, followed by maintenance therapy: dietary changes with increased fibre and clear sugar-free fluids; lactulose as maintenance*).

The good candidate additionally addresses psychosocial issues comprehensively and establishes a *clear follow-up plan, with possible involvement of a dietician and specialist paediatric services if poor response to this intervention*. This candidate also uses this visit as an opportunity for *health promotion (deworming) and discusses testing for other underlying problems, for example, hypothyroidism.*

### Role play – Instructions for actor

Adult female. Calm. 20–35 years old

Opening statement:

Hello Doctor. I brought my child because she is leaking stools for the last week, without feeling that she needs to defecate. I’m very worried about this … none of my other children had this problem. I had to put her back onto the nappy to protect her clothes.

Open responses: Freely tell the doctor …

This child is your youngest. She is 3 years old. Her siblings are 7 and 11 years old. She is a healthy child, but you noticed her appetite going down in the last few weeks.

Closed responses: Only tell the doctor if she or he brings this up:

- She doesn’t know when the leaking will come. It is like dark brown liquid with a strong faeces smell. It happens about 2–3 times per week. You have never seen blood, and it does not have mucus in it.
- The child had very hard and painful stools for about 2 months now. She cries when she must pass stool, and often will only pass one or two small pieces. She had normal stools before.
- You tried an over-the-counter laxative before she started leaking, but got scared when the leaking started again. You think you did some damage …
- Her appetite is not good – she used to be a good eater, but this stopped in the last few weeks. She now eats a little bit, then says she doesn’t want anymore.
- She eats mostly porridge in the morning, then bread at lunch-time at the day-care centre, and will have a little bit of food at home … a few pieces of chicken with some rice. She doesn’t like fruit or vegetables. She drinks a lot of diluted apple juice.
- You breastfed her exclusively for 4 months. All her immunisations are up to date, and at her last visit, she was growing well.

#### Your medical history

This child has no medical history of note. Her birth and pregnancy were normal. She grew and developed normally and has received all her immunisations to date.

#### Ideas, concerns and expectations

You’re very worried that you caused some damage with the laxative, and now she is leaking and may need to have surgery to fix her.

### Patient’s notes/examination findings

- Healthy, happy, playful child
- Well nourished.
- No red flags in *Road to Health* book, as per triage nurses. *Road to Health* book not available now.

#### Vitals

- Heart rate: 123/min. Easily palpable pulses.
- Temperature: 36.5 °C
- Random haemoglucotest (HGT): 6.7 g/dL
- Ward Haemoglobin: 14.3 g%

#### Examination

- Skin, hair, mucosae: All normal
- No suggestion of cardiac or respiratory problems
- No neurological abnormalities
- Abdominal examination: Protuberant abdomen, although not grossly distended. Non-tender to superficial palpation, and allows deeper palpation, with no organomegaly, but some mild discomfort. Bowel sounds are soft.
- Anus: Tender, spastic sphincter. Patient does not allow examination.
- Urine dipstick test: No abnormalities detected
- X-rays and blood tests: Not done

